# Creaming behavior prediction of argan oil in water emulsion stabilized by lacto-fermentation: creaming index

**DOI:** 10.1186/s12896-021-00711-9

**Published:** 2021-09-18

**Authors:** Soumaya El Bouchikhi, Philippe Pagès, Azeddine Ibrahimi, Yahya Bensouda

**Affiliations:** 1 Laboratory of Pharmaceutics, Faculty of Medicine and Pharmacy, Mohammed V University, Rabat Instituts, BP 6203 Rabat, Morocco; 2 Laboratory of Medical Biotechnology, Faculty of Medicine and Pharmacy, Mohammed V University, Rabat, Morocco; 3 PhP Stats, Création et analyse d’information, Conseil, études et formations en statistique, 19, rue Pasteur, 94170 Le Perreux, France

**Keywords:** Emulsion O/W, Argan oil, Sodium caseinate, Starch, Dairy-like products, Lacto-fermentation, Creaming index

## Abstract

**Background:**

In order to improve the taste acceptability of certain nutritional oils, it has been decided in this study to introduce them in an emulsion whose surfactant is casein, then to carry out a lacto-fermentation, leading to a dairy-like product with added nutritional value and health benefit. In this context, a plan of mixtures has been proposed for the preparation of emulsions based on argan oil, sodium caseinate and starch, with concentrations ranged between (10–20%) and (0–2%) and (0–1.5%) respectively. All emulsions were homogenized at two high stirring velocities (10,000–20,000 rpm) and two stirring times (5–20 min). The physical stability was assessed by visual analysis and microstructural measurements. The Creaming index was calculated for selected emulsions to predict their creaming behavior.

**Results:**

All emulsions showed a creaming behavior except one emulsion that required the highest values of all factors, which showed the highest creaming index with an average particle size of 11.27 μm. The absence or the variation of one or all factors led to various degrees of instabilities verified in all other emulsions. Due to the synergistic action of all parameters, the emulsion stability was attributed to the reduction of droplets size, the increase of continuous phase viscosity and the decrease of coalescence.

**Conclusion:**

The parameters that played a major role in the stability of the emulsion consists of: stirring velocity and time, sodium caseinate/oil ratio and starch/sodium caseinate ratio. The underlying structure and the interaction of the fluid droplets within the solid like product is what holds the stability of the product against settling or separation during fermentation.

## Background

During the last decade, fundamental studies opened a new field of research dealing with the health promoting features of so called functional foods [1]. Argan oil is considered one of the most important seasoning oils that provide a health benefit beyond its basic nutritional functions. This oil represents a rich source of linoleic and oleic acids 37% and 45% respectively, in addition to other compounds (tocopherols, polyphenols, sterols, carotenoids, xanthophylls and squalene) [2]. The unsaponifiable fraction is very abundant in these compounds and has a powerful antioxidant effects as proved on low-density lipoprotein (LDL) isolated from human plasma [3]. The consumption of dietary Argan oil has proved to have a positive effect in the prevention of certain diseases such as high cholesterol [4], heart disease [5], diabetes [6] that are known for having a high incidence in human health. Moreover it has an ameliorative effect on skin elasticity and hydration on postmenopausal women population [7, 8]. All these beneficial effects required a minimum daily amount of 20 g of argan oil. However its taste acceptability remain an important factor that limits its consumption worldwide.

To improve the acceptability of such a functional product, it is proposed to introduce it into an innovative dairy-like product having both a pleasant taste and a positive impact on health. The development of such a product is done in two stages: 1. Formulation of a stable emulsion at 20% of argan oil. 2. Its lacto-fermentation using lactic acid bacteria *Strep.thermophilus* and *Lactobacillus delbrueckii ssp Bulgaricus*. This last step assumes the presence of lactose and casein in the emulsion. The ferment allows a natural acidification of the medium by transformation of lactose into lactic acid. This condition of acidic pH allows the aggregation of casein to reach the gelled state, which is appreciated in fermented dairy products. The formulation of the emulsion could require either the search for the critical value of the hydrophilic lipophilic balance (HLBc), or the search for the zero value of the hydrophilic lipophilic difference (HLD). However, a more direct method of applied research was adopted, the experimental designs, in which different composition and process factors were integrated. Among the mixing factors, it was considered those involved in the stabilization mechanism of oil-in-water emulsions at the external, interfacial and internal level: the amount of modified starch as stabilizer for the external phase, the amount of sodium caseinate as emulsifier for the interface [9], and the quantity of argan oil as functional ingredient for the internal phase.

The stabilizers are normally biopolymers such as proteins or polysaccharides that confer long-term stability to the emulsion, by maintaining the often-ephemeral dispersion authorized by the emulsifier. They act by the adsorption mechanism or simply by modifying the viscosity of the aqueous phase [10, 11]. Emulsifiers are small surfactant molecules, which have the ability to stabilize emulsions in a short time by reducing the tension at the oil/water interface and the film formation on the surface of the oil [12, 13]. Most hydrocolloids act as oil stabilizers in water emulsions, but only a few acts as emulsifiers such as modified starches. In fact, their chemical modifications increase their hydrophobicity and allow them to show a strong surface activity [14, 15]. Heating in situ can further modify the properties of emulsions stabilized by starch granules [16]. In fact, when heated in an aqueous medium, the starch gels. This gelatinization process includes swelling of the starch granules, leakage of amyloidosis of the granules, loss of molecular and crystalline order; which increases the viscosity of the external phase and thus has a positive impact on the stability of the emulsion [17, 18]. Concerning the process factors, we have retained the speed and the duration of stirring. The effect of high shear homogenization on the physical properties of the oil in water emulsion is dependent of the speed applied to the homogenizer; Which is effective in improving the physical stability of emulsion, by promoting reduction of droplet diameter [19].

The purpose of this study was to formulate a 20% sodium caseinates-stabilized oil in water emulsion, in which both stability towards coalescence and a controlled destabilization was needed. Then to carry out its lacto-fermentation, leading to an acid gel-network capable of holding the dispersed oily phase with an optimal stability. A preliminary work had been conducted and demonstrated the influence of milk proteins and starch on syneresis in a mixed model system and determined their effective concentrations [20]. Accordingly, an experimental design was proposed by using, on a basic matrix consisting of 5% lactose, 3.5% milk proteins and qs 100 g of water, a mixture design of the following selected factors: argan oil (10–20%), sodium caseinate (0–2%) and starch (0–1.5%) as well as stirring velocity (10,000–20,000 rpm) and stirring time (5-20 min). The effects of these formulation variables on the creaming behavior were evaluated through physical stability and morphological properties of all emulsions. The most stable ones were then fermented and evaluated through the macroscopic observation of the apparition of a thick cream layer, indicating phase separation, after fermentation process.

## Results

In emulsions, the underlying structure and the interaction of the fluid droplets are what hold the stability of the product against settling or separation. Phase separation results in an instability called creaming, which is ultimately caused by flocculation, coalescence, and gravitational separation [21]. The results of the experimental design are reported in Table 1, all factors have been combined in a single column in the following order, (A) Oil/(B) Starch/(C) protein (sodium caseinate)/(D) Stirring velocity/(E) Stirring time with (m) for their minimum value, (M) for their maximal value and (a) for the average value.

**Table 1:** Mean droplets size and size variation

Samples	Factors combination m: minimal value M: maximal value a: average value Oil/Starch/Protein/StirringV/StirringD	Responses				
		1	2	3	4	5
		Creaming behavior	Size at t0	Size at t5	St5 − St0	Size Var
		1/yes to 5/no	= S0 (μm)	= S5 (μm)	(μm)	(%)
1	m/m/m/M/m	2	15.87	28.56	12.69	79.96
2	a/a/a/a/a	2	29.34	42.17	12.83	43.72
3	M/m/M/m/M	1	58.32	67.04	8.72	14.95
4	M/m/m/m/m	1	55.62	115.4	59.78	107.48
5	M/m/M/M/m	2	20.52	24.8	4.28	20.86
6	M/M/m/M/m	2	12.58	20.62	8.04	63.91
7	m/M/M/m/m	1	56.22	66.65	10.43	18.55
**8**	**m/M/m/M/M**	**3**	**11.41**	**12.49**	**1.08**	**9.46**
9	a/a/a/a/a	2	23.6	31.96	8.36	35.42
**10**	**m/M/M/M/m**	**4**	**10.9**	**11.67**	**0.77**	**7.06**
**11**	**M/m/m/M/M**	**3**	**14.01**	**15.63**	**1.62**	**11.56**
12	m/m/M/m/m	1	64.82	77.82	13	20.05
13	m/M/m/m/m	1	48.14	65.65	17.51	36.37
14	m/M/M/m/M	2	20.71	25.25	4.54	21.92
15	M/M/M/m/m	1	21.8	30.34	8.54	39.17
16	a/a/a/a/a	2	26.45	33.17	6.72	25.40
17	M/M/m/m/M	2	24.77	31.06	6.29	25.39
**18**	**m/m/M/M/M**	**3**	**12.66**	**13.79**	**1.13**	**8.92**
19	m/M/m/M/m	1	22.23	43.39	21.16	95.18
20	M/M/m/m/m	1	31.24	45.66	14.42	46.16
21	M/m/m/M/m	2	12.81	35.82	23.01	179.62
22	M/m/M/m/m	1	59.69	108.19	48.5	81.25
23	m/m/m/m/M	1	15.16	35.7	20.54	135.49
**24**	**M/M/M/M/M**	**5**	**11.24**	**11.27**	**0.03**	**0.27**

Phase separation and Variation of mean droplets in emulsions after 5 h of storage according to the 24 combinations proposed by the Design Expert: minima (m), maxima (M) and the average (a) of the different factors. In bold: selected emulsions. Creaming behavior classification: 1: separation; 2: visible separation; 3: slight separation; 4: non visible separation; 5: no separation

The Expert Design analysis consists on the finding of the best power transformation to linearize the results. After transforming the power using box-cox diagrams, the design expert draws the Pareto chart of different factors with the Bonferroni and the t-value limits. These graphs make it possible to visualize the amplitude of the influences of the studied factors. Accordingly, the droplets size of emulsions was analyzed, the creaming behavior was evaluated, and the creaming index of selected emulsions was calculated.

### Microstructure and droplet size of emulsions evaluation

The development of the microstructure of the designed emulsions has been studied and a correlation between droplets size and formulations parameters has been proposed. The study considered the droplet size at t0, the droplet size 5 h later at t5 and the variation rate of droplets size. The Box-Cox method of the Design Expert recommended the optimal power transformed to the linear equation for each case (Table 2). The significance tests were valid and it was not necessary to transform the response to improve its modeling.

**Table 2:** Linearization of size analysis after powers transforms by the Box-Cox method

	Droplets size at T0	Droplets size at T5	Rate size Variation
Box-Cox for power Transform	Log_10_	√	√
R^2^ brut	0.96	0.96	0.93
R^2^ adjusted	0.93	0.93	0.88
R^2^ Predicted	0.85	0.8	0.74
Constant	1.34	5.81	5.72
A	0.0087	0.027	− 0.29
B	− 0.065	− 0.73	− 0.96
C	0.026	− 0.15	− 1.6
D	− 0.21	− 1.62	− 0.96
E	− 0.072	− 0.89	− 1.51
AB	− 0.055	− 0.55	
AE	0.079	0.33	− 0.9
BC	− 0.067		0.68
BD		0.41	
DE			− 0.66
CE	0.031	0.31	
DE	0.047		− 0.94

Regression coefficients (R^2^), and coded factors (varying between + 1 and − 1) of the linearization equation for parameters: A (oil), B (starch), C (protein), D (stirring speed) and E (stirring time)

**Fig. 1:**
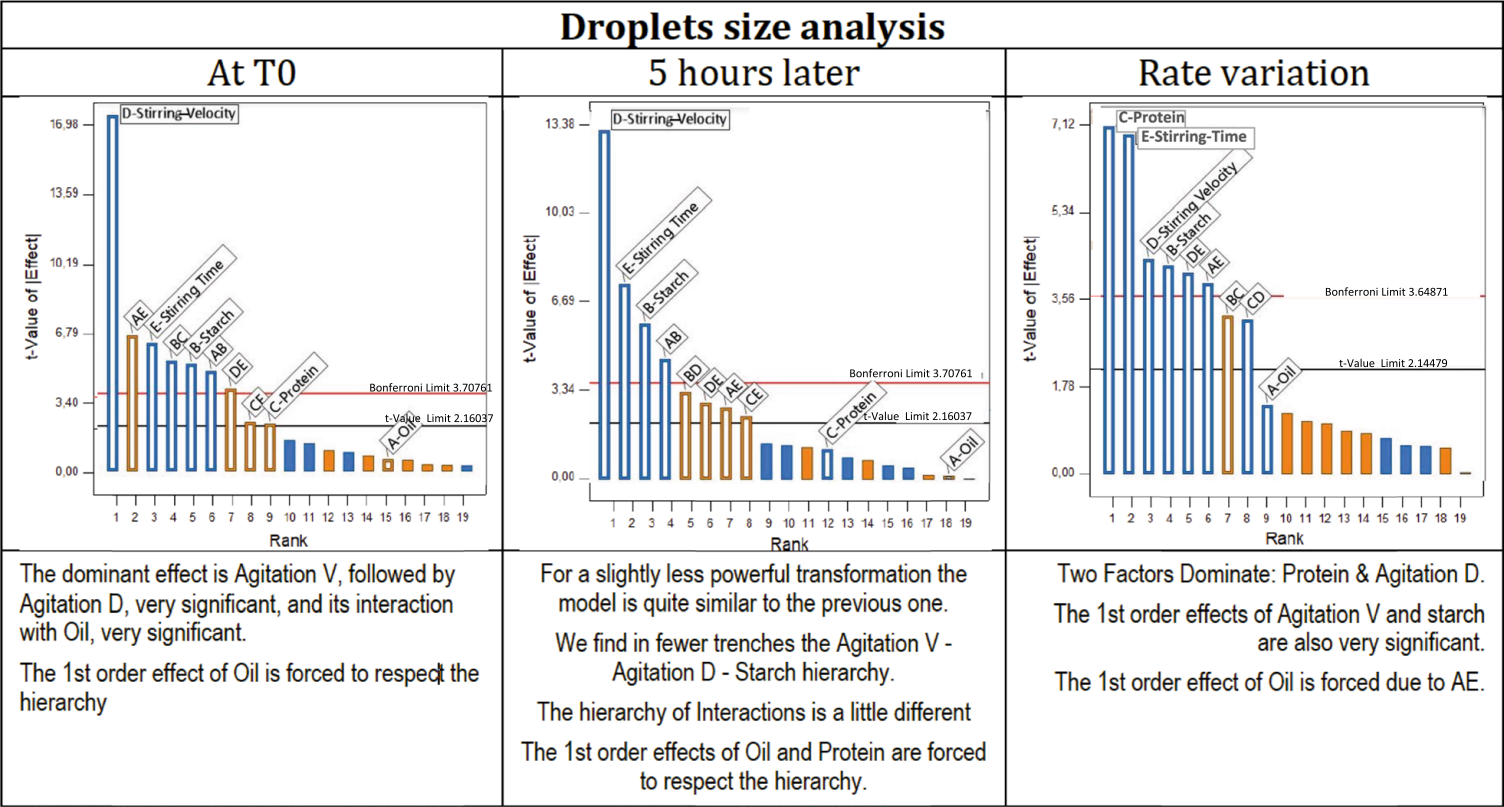
Pareto Chart for the droplets size analysis

**Fig. 2:**
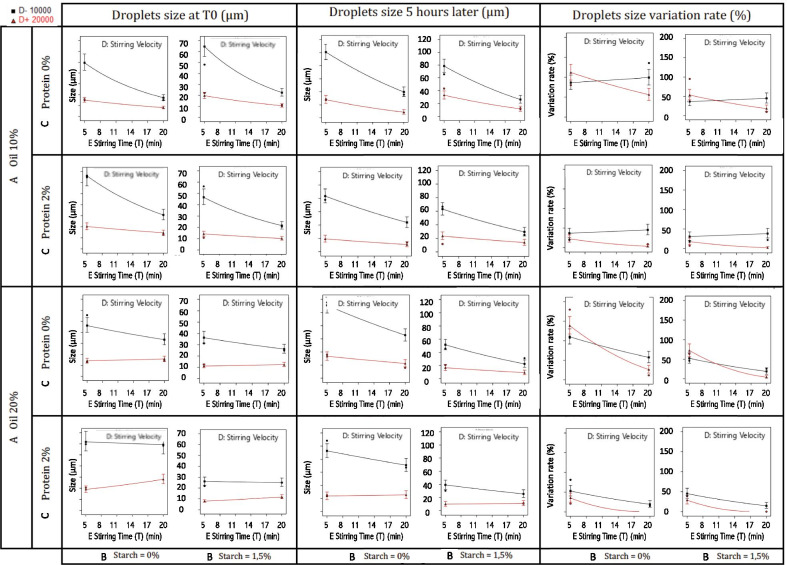
Droplets size values at T0, 5 h later T5, and their variation rate according to contents (% of oil, Starch and protein) and process (stirring velocity and stirring time)

The Pareto graphs show the degree of domination of different parameters in the proposed linearization models (Fig. 1). The five factors (A: Oil; B: Starch; C: Protein; D: Stirring Velocity; E: Stirring Time) are above the Bonferroni limit, exhibiting their influence on the variation rate of droplets size. The droplets size after emulsion preparation at t0 and five hours later at t5, as well as the rate of their variation over this period of time, were provided by the design expert and reported in graphs (Fig. 2)**.** These later shows the combined influence of all parameters including the ingredients and the stirring conditions at their minimum and their maximum level on the droplets size variation, which is a great indicator of the stability of emulsions.

### Creaming behavior evaluation

The creaming behavior of the emulsions was evaluated for emulsions before fermentation and then for fermented emulsions.

#### Emulsions

**Fig. 3:**
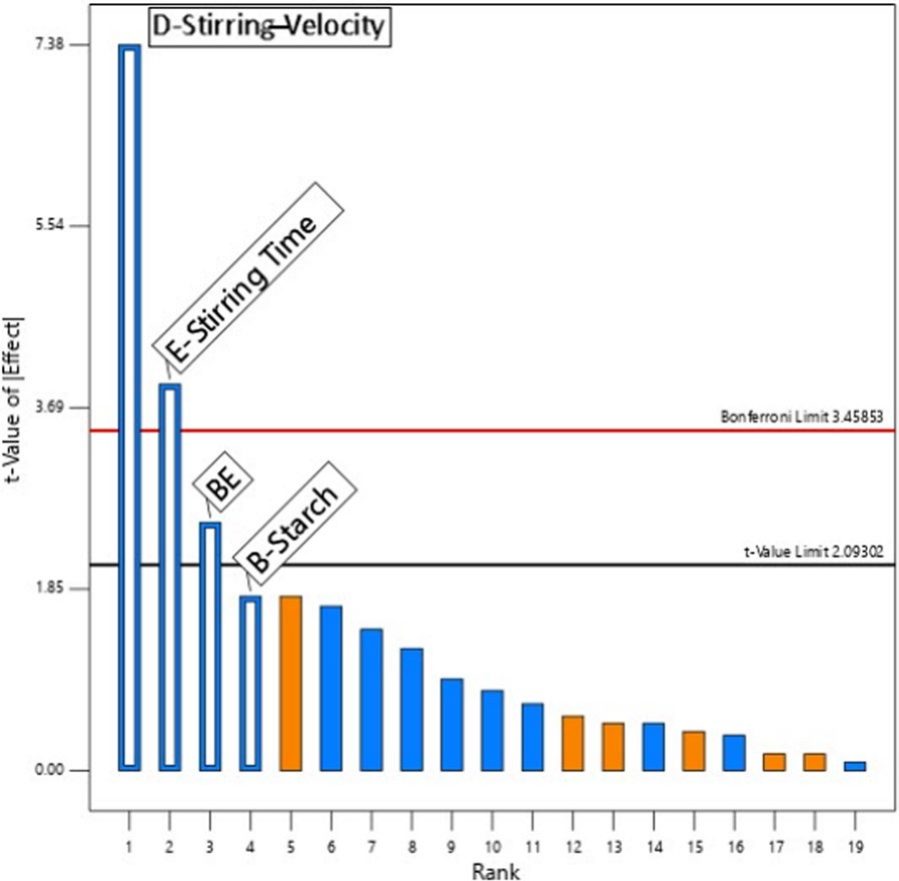
Pareto chart for creaming behavior analysis

**Fig. 4:**
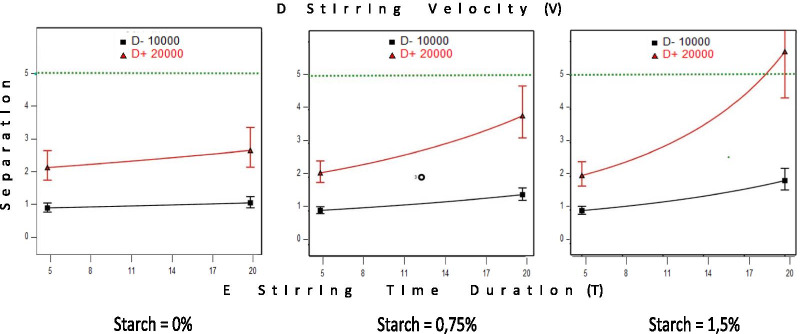
Creaming analysis by DoE showing the optimum at high values of the 3 influencing factors: starch % and stirring time (T) and stirring velocity (V)

For the study of creaming, the Box-Cox method recommends a transformation 1/square root (Fig. 3). The following analysis is performed with this transformation; it emerges from the Pareto-chart that all significant effects are negative. The dominant effect is stirring velocity (D), followed by the important effect of stirring time (E), and the significant effect of the interaction of agitation duration with starch. The first order effect of Starch is forced to respect the hierarchy. The creaming behavior of all samples was observed at room temperature 5 h later of their preparation. The emulsions were classified from level 1 (fast separation) to level 5 (no separation) as shown in Table 1. All emulsions displayed rapid creaming except five samples: S8; S10; S11; S18 and S24. A visible thick cream layer and a lower serum phase were clearly observed when the speed and the duration of homogenization were not at their highest level, and when no starch and/or sodium caseinate were added. According to our results, the DoE revealed that the most influent factors on emulsion’s stability were the stirring velocity, the stirring time duration and the starch rate (Fig. 4).

#### Lacto-fermented emulsions

The five selected emulsions were fermented directly after their preparation to evaluate their stability after fermentation (6.7–4.6) at 42 C: All emulsions showed a creaming behavior during the fermentation process except emulsion S24.

### Creaming index calculation

**Fig. 5:**
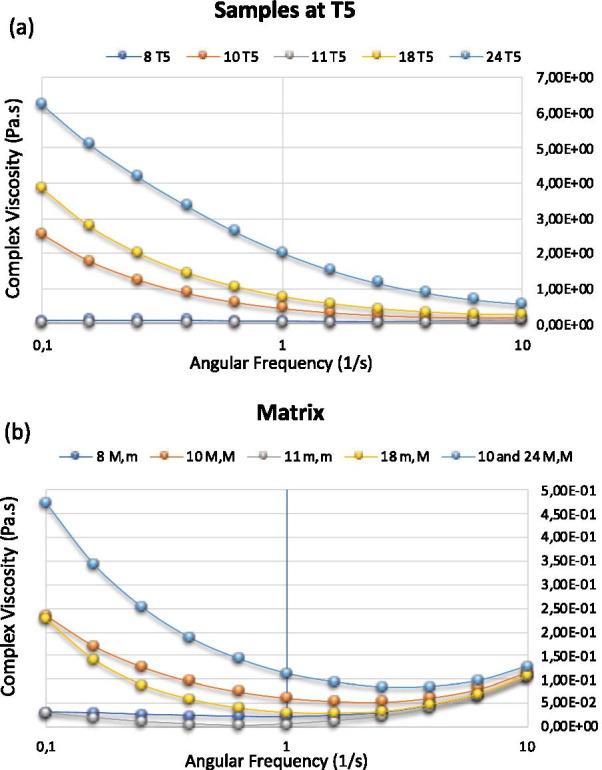
Rheological behavior of the 5 selected emulsions (**a**) and their matrix (**b**), which contains starch, Caseinate Na at their maximal (M) or minimal (m) values

This parametric selection above could be completed by a pharmacotechnical analysis, which can reveal the influence of new factors such as the rheological behavior of matrix and emulsions, as well as the static flow threshold or the Yield value that are related to the micro-pressure of free droplets or their flocculated clusters (Fig. 5). The basic matrix contains a mixture of milk proteins and lactose, to which varying amounts of starch and sodium caseinate may or may not be added. These compositions are capable of giving a more or less pronounced non-Newtonian rheological behavior of the plastic type. This is particularly characterized by a static flow limit that is the minimum stress from which the strain begins. In the absence of external stress, the suspended elements should exceed this threshold by their own weight before the matrix deforms.

## Discussion

### Microstructure and droplet size

The results reveled a large variation of the mean droplets size in all emulsions after their preparation t0 and 5 h later t5, as well as in their droplets size variation rate as shown in Table 1. Sodium caseinate percentage and the duration of homogenization had the dominant influence on the droplets size variation rate followed by the velocity and the starch percentage. In this research, sodium caseinates and hydroxypropyl distarch phosphate (HPDSP) were added to emulsions as emulsifier and stabilizer. According to previous publishes studies, sodium caseinate-based emulsions have long-term stability as its components readily adsorb onto the interface of oil droplets, which provide them protection against coalescence at neutral pH via a combination of electrostatic and steric stabilization [22–24]. Hence, the magnitude of the depletion force in a caseinate-based emulsion depends on the relative dimensions of the oil droplets, which is related to the manufacturing process [25]. The smallest droplets variation rates were 0.27%; 7.06%; 8.92%; 9.46%; 11.56%, which were registered for the following emulsions S24; S10; S18; S8; S11 respectively.

Of the 5 emulsions retained, only S11 and S24 are 20% oil and therefore have the largest droplet surface or oil/water interface to be covered. According to Dickinson and al, the regime with no creaming and droplet flocculation is observed when sodium caseinates concentration is sufficient for nearly saturation coverage of the droplets of emulsion [26]. The smallest size variation rate 0.27% was observed for the emulsion S24، which showed no creaming behavior and where all factors were at their high levels. Emulsion S11 exhibited the greatest variation size ratio and a strong creaming behavior even though the stirring conditions were at their highest level, which could be explained by the absence of sodium caseinate and starch [27]. Emulsions S10 and S8 and S18 exhibited intermediate variation size rates and showed a slight phase separation. Destabilization in emulsions S18 and S10 could be related to depletion flocculation due to the presence of an excess of unabsorbed sodium caseinate that induced de-flocculation (10% O/W and 2% of sodium caseinate). Therefore the low size variation rate of these emulsions is due to saturation coverage of droplets that was able to prevent any further coalescence [28]. In fact, the stability of oil-in-water emulsions prepared with sodium caseinate is particularly sensitive to the concentration of the protein emulsifier for a constant fraction of oil. At low concentrations of sodium caseinate bridging-flocculation may occur. Conversely, at high concentrations of sodium caseinate, oil droplets may also lose their stability through a depletion-flocculation mechanism [29, 30]. These two types of flocculation, bridging or depletion are readily distinguished in practice. Only strong shear forces can disrupt the clusters of droplets held together by protein bridges, whereas flocks arising from depletion are re-dispersible by gentle stirring or dilution. In emulsion S8 where no sodium caseinate was added, hydroxypropyl distarch phosphate (HPDSP) was at its highest rate 1,5%, which increased the viscosity of the outer phase, slowing down phase separation and therefore had a positive impact on emulsion stability.

Considering the above, It could be concluded that the addition of sodium caseinate at its optimal concentration 2% ensured a complete coverage of oil droplets of the disperse phase of the emulsion at 20% O/W, which generate an electrostatic repulsion between particles, which stabilized the emulsion and prevented coalescence. Furthermore, the addition of starch at 1.5% increased the continuous phase viscosity reducing the creaming velocity of oil droplets in emulsions slowing down phase separation. Therefore the combination of both ingredients at their optimal concentrations is essential for ensuring the stability of the emulsion at a certain concentration of the disperse phase and for given stirring conditions. The nature of aqueous phase components, the concentration of disperse phase and the size of emulsion droplets play a major role in the stability of emulsions.

### Creaming behavior

The creaming behavior observed during the stability study of the emulsion occurs during lacto-fermentation process, it is accentuated by the phenomenon of casein aggregation. During the acidification process, the pH is lowered toward the isoelectric point of the proteins, the inter-droplet interaction changed from a repulsive to a net attraction. Which induced droplet flocculation, causing a self-association of the adsorbed and non-adsorbed protein components, and transformed the sodium caseinate-stabilized emulsion into an aggregated emulsion gel [24, 31]. The addition of hydroxypropyl distarch phosphate (HPDSP) increases the gel strength and viscosity and reduce syneresis, which may be indicative of faulty fermentation and therefore may be controlled [17, 32]. The interaction between casein and HPDSP in yoghurt system has been explored previously, the casein micelles having a net positive charge and the HPDSP, being negatively charged, are assumed to bind via electrostatic forces. Therefore the surface of both casein aggregates and starch micelles become positive which should induce electrostatic repulsion between them and stabilize the system. It was also reported that the interaction between casein and HPDSP in yogurt system was also the result of “steric stabilization”: blocks of low affinity areas on the casein molecules may protrude from the surface of the starch micelles, a mechanism termed ‘steric stabilization’. Both the “electrostatic repulsion” and “steric stabilization” contribute to the stability of the system [33, 34]. Therefore, the underlying structure and the interaction of the fluid droplets within the solid like product is what holds the stability of the product against settling or separation.

### Creaming index

In the case of the 5 selected emulsions, 4 types of matrices were mentioned (a common one between S10 and S24), which gave us at the minimum values of the angular frequency, the value of the static flow limit of each matrix or the Yield value as shown in Fig. 5a. It was mentioned that this limit is more marked for the common matrix to emulsions S10 and S24 followed by the one of emulsion S18 and finally the ones of emulsions S8 and S11. Moreover, the stress of the dispersed elements corresponds to that of the droplets in suspension. The reasoning consists in calculating the micro-stress of the dispersed element and to compare it with this threshold. Table 3 shows the 5 emulsions’ values of the static flow limits of the matrices, the average size of the droplets and those of the largest droplets followed by their micro-constraints. These being lower than the static flow limits; the creaming index was then calculated. This number represents the minimum number of droplets that an aggregate must contain to overcome the static flow limit by its weight. Therefore, it causes the deformation and the migration of the droplets. It was calculated for the medium size and for the largest droplet sizes. It is thus expressed in an interval and represents the necessary number of droplets per aggregate to cause the creaming. The higher this index, the more stable the emulsion. Accordingly, emulsion 24 is the most stable of all emulsions since it has the highest creaming index.

**Table 3:** Droplets size, yield value and creaming index

Sample	8	10	11	18	24
Oil-Starch-Casein-SV-ST	m-M-m-M-M 10-1.5-0-M-20	m-M-M-M-m 10-1.5-2-M-5	M-m-m-M-M 20-0-0-M-20	m-m-M-M-M 10-0-2-M-20	M-M-M-M-M 20-1.5–2-M-20
Yield value (Pa s)	3.01E−01	2.35E+00	2.96E−01	2.28E+00	4.74E+00
Average droplet size-ADS (μm)	12.49	11.67	15.63	13.79	11.27
Largest droplet size-LDS (μm)	34.234	34.25	58.75	33.89	20
ADS Pressure (Pa s)	4.03E−04	3.80E−04	5.09E−04	4.49E−04	3.67E−04
LDS Pressure (Pa s)	1.11E−03	1.12E−03	1.91E−03	1.10E−03	6.51E−04
Creaming index for ADS (Number)	747	6.183	581	5.076	12.914
Creaming index for LDS (Number)	270	2.107	155	2.066	7.277

SV: Stirring velocity; ST: Stirring time (duration); m: minimum; M: Maximum; ADS: Average Droplet Size; LDS: Large Droplet Size

## Conclusion

According to the findings, the velocity and the duration of homogenization, sodium caseinate/oil ratio and starch/sodium caseinate ratio are the main parameters that played the major role in the stability of the final product. The emulsion S24 showed the highest creaming index and did not exhibit any creaming behavior during its fermentation process at 42 C. Thus, it can be considered as the most stable formulation for use as functional food. Therefore, Understanding the sensory attributes that characterize the effective ideal product is of great interest to optimize the formulation.

## Methods

### Ingredients

Sodium caseinates, Starch: Hydroxypropyl distarch phosphate (E1442), Milk proteins and lactose, all provided by Trade Bio-Industries Morocco; Lactic acid bacteria: *Strep.thermophilus* and *Lactobacillus delbrueckii ssp Bulgaricus (*commercial yogurt starter culture freeze-dried)*.* Argan oil was provided by Association ibn al baytar, Rabat. Freshly distilled water was used for preparation of all samples.

### Equipment

Design Expert 10 software edited by Stat-Ease inc; Thermostatic Water bath; High Shear Homogenization (TissueRuptor II (230 V, 50/60 Hz, EU/CH) Qiagen 9002756); Optical microscopy (Nikon microscope Eclipse LV100ND, Tokyo, Japan); Software Image J 1.52 a (http://imagej.nih.gov/ij); Rheometer MCR 500 (Physica, Germany).

### Experimental design

An experimental design was selected to define the formulation space for emulsions mixtures. Starch, Protein (= Sodium caseinate), Oil phase, Speed and Time of homogenization were used as independent variables. The morphological parameters of emulsions (mean droplet size and variation size rate) were selected as dependent variables. In this experimental context, the expert design software offers an optimal plan of 24 experiments including 3 at median values as indicated in Table 4.

**Table 4:** The experimental design

Block	Run (samples)	Space type	Composition factors			Process factors		Abbreviation by minima (m), Maxima (M) and average (a) values
			A: Oil	B: Starch	C: Protein	D: Stirring velocity	E: Stirring duration	
			%	%	%	Rpm	Min	
Block 1	1	Vertex	10	0	0	20,000	5	m, m, m, M, m
Block 1	2	Center	15	0.75	1	15,000	12.5	a, a, a, a, a
Block 1	3	Vertex	20	0	2	10,000	20	M, m, M, m, M
Block 1	4	Vertex	20	0	0	10,000	5	M, m, m, m, m
Block 1	5	Vertex	20	0	2	20,000	5	M, m, M, M, m
Block 1	6	Vertex	20	1.5	0	20,000	5	M, M, m, M, m
Block 1	7	Vertex	10	1.5	2	10,000	5	m, M, M, m, m
Block 1	8	Vertex	10	1.5	0	20,000	20	m, M, m, M, M
Block 2	9	Center	15	0.75	1	15,000	12.5	a, a, a, a, a
Block 2	10	Vertex	10	1.5	2	20,000	5	m, M, M, M, m
Block 2	11	Vertex	20	0	0	20,000	20	M/m/m/M/M
Block 2	12	Vertex	10	0	2	10,000	5	m/m/M/m/m
Block 2	13	Vertex	10	1.5	0	10,000	5	m/M/m/m/m
Block 2	14	Vertex	10	1.5	2	10,000	20	m/M/M/m/M
Block 2	15	Vertex	20	1.5	2	10,000	5	M/M/M/m/m
Block 2	16	Center	15	0.75	1	15,000	12.5	a/a/a/a/a
Block 3	17	Vertex	20	1.5	0	10,000	20	M/M/m/m/M
Block 3	18	Vertex	10	0	2	20,000	20	m/m/M/M/M
Block 3	19	Vertex	10	1.5	0	20,000	5	m/M/m/M/m
Block 3	20	Vertex	20	1.5	0	10,000	5	M/M/m/m/m
Block 3	21	Vertex	20	0	0	20,000	5	M/m/m/M/m
Block 3	22	Vertex	20	0	2	10,000	5	M/m/M/m/m
Block 3	23	Vertex	10	0	0	10,000	20	m/m/m/m/M
Block 3	24	Vertex	20	1.5	2	20,000	20	M/M/M/M/M

Five quantitative factors at 2 levels allowing the Design Expert to generate 21 "matrix" tests and 3 points in the center, all divided into 3 blocks. These 21 "matrix" tests are sufficient to estimate the constant, the two degrees of freedom of the block effect, the 5 1st order coefficients and the 10 2nd order interactions under satisfactory orthogonally conditions (the VIF do not exceed 1.14). This is likely an optimal plan

### Preparations of emulsions samples

Oil in water emulsions were prepared by phase inversion at constant concentrations of milk proteins 3,5% and lactose 5% with different final concentrations of argan oil, sodium caseinates and starch ranged between (10–20%) and (0–2%) and (0–1.5%) respectively and qs 100 g of water. The concentrations of sodium caseinate and starch were suggested based on our previous study [20]. Milk proteins, lactose, sodium caseinate and starch were dissolved in distilled water under magnetic agitation, heat treated to allow starch gelation and pasteurized at 85 °C for 15 min. Then cooled to 45 °C before homogenization with argan oil in a water bath fixed at 45 °C to form emulsions. These later were prepared at two stirring velocities (10,000–20,000 rpm) for two time durations (5–20 min). All emulsions were prepared freshly before being evaluated. The most stable emulsions were fermented by lactic acid bacteria and then assessed.

### Evaluations of the emulsions stability

#### Creaming behavior

The emulsion stability was first evaluated through the macroscopic observation of the apparition of a thick cream layer during their 5 h of storage at room temperature (phase separation). The emulsions showed different creaming behaviors at different times of their storage going from fast separation to no separation (1–5) as indicated in Table 1.

#### Optical microscopy and image analysis

The morphological properties in terms of droplet diameter and dispersion were characterized using an optical microscopy. The emulsions were examined by the measurement of the droplets size after preparation (T0), and 5 h later (T5). Immediately after preparation, 35 mL of each emulsion was poured into a cylindrical tube sealed with a plastic cap and stored at 25 C for a period of 5 h. After storage, the majority of emulsions were separated into a top cream phase (T5top) and a bottom serum phase (T5bottom). The microstructures of all formulations were analyzed. A drop of every emulsion was transferred to a glass slide covered by a cover slip, and evaluated at an optical microscope coupled with a software for image analysis (Image J 1.52 a): Three images were randomly taken for each distribution, and observed at a magnification of 40× and 100×. For each image 50 droplets were counted to obtain the droplet size distributions. The image analysis provided a droplet size distribution in terms of pixels. The pixel-scale values were converted into microns by a scaling factor, and the calibration to transform pixels to actual size (μm) was given by the full width of an image measured [35]. The parameters used as criteria to select the optimal model included the average droplet size (d32) and the variation size rates after 5 h of storage (T5 − T0). The average droplets size was measured by means of the software and the volume–surface mean diameters (d32) of the emulsions [36].

#### Rheological testing

To characterize the rheological properties in the liquid state, small amplitude oscillatory tests were performed on a MCR 500 (Physica, Germany) rheometer equipped with a couet system. The measurements were carried out using a cylindrical geometry with 35 mm of diameter at room temperature using frequency sweeps between 0.05 and 500 Hz at a strain of 5% (linear visco-elastic regime). The dynamic viscoelastic properties were measured for selected emulsions, 15 ml of the top of the emulsions were poured into a walled concentric cylinder consisting of an inner rotating acrylic cylinder to evaluate the creaming behaviour of each sample after their preparation and 5 h later. Flow curves were plotted for emulsions and their continuous phases. The parameters used as criteria to select the optimal model included: dynamic storage (G^0^), loss modulus (G^00^), complex modulus (G^*^) and apparent viscosity. All measurements were performed in triplicate at 25 °C.

#### Data analysis

All statistical analyses were performed using the Design Expert version 10, a statistical software package from Stat-Ease Inc that is specifically dedicated to perform design of experiments (DoE). In order to reduce scattering effects and to compare the samples, all physical results had been normalized. The Residual analysis, the coefficient of determination (adjusted R^2^), the significance of the models and the lack of fit were used to check the quality of the model. The robustness of the models was evaluated by determining the squared correlation coefficient (R^2^) for predicted versus measured values in cross-validation. In addition to the ratio of standard deviation to root mean square error of calibration.

### Lacto-fermentation of selected emulsions

The most stable emulsions, having a droplet size variation after 5 h of storage (T5 − T0) of less than 12% and a separation index > or = 3, were selected (They were subjected to the evaluation of their continuous phase viscosity, before their fermentation) then fermented with *lactobacillus bulgaris* and *streptococcus thermophillus* at 42 C. The fermented emulsions were stored at 4 C.

## Data Availability

The datasets used and/or analyzed during the current study are available from the corresponding author on reasonable request.

## Abbreviations

DoE: Design of experiments
LDL: Low-density lipoprotein
HLBc: Critical hydrophilic lipophilic balance
HLD: Hydrophilic lipophilic difference
HPDSP: Hydroxypropyl distarch phosphate
